# Supplementary material for: A longitudinal resource for studying connectome development and its psychiatric associations during childhood
Source: Sci Data. 2022 Jun 14;9:300. doi: 10.1038/s41597-022-01329-y (PMC9197863; doi:10.1038/s41597-022-01329-y)
Supplement: Supplementary file 1 — Supplementary File [file 41597_2022_1329_MOESM1_ESM.pdf]

Table of Contents

Supplementary Figures 2
Supplementary Figure 1 2
Supplementary Figure 2 4
Supplementary Figure 3 5
Supplementary Figure 4 6
Supplementary Figure 5 7
Supplementary Figure 7 8
Supplementary Figure 8 8
Supplementary Tables 9
Supplementary Table 1 9
Supplementary Table 2 9

Supplementary Figures

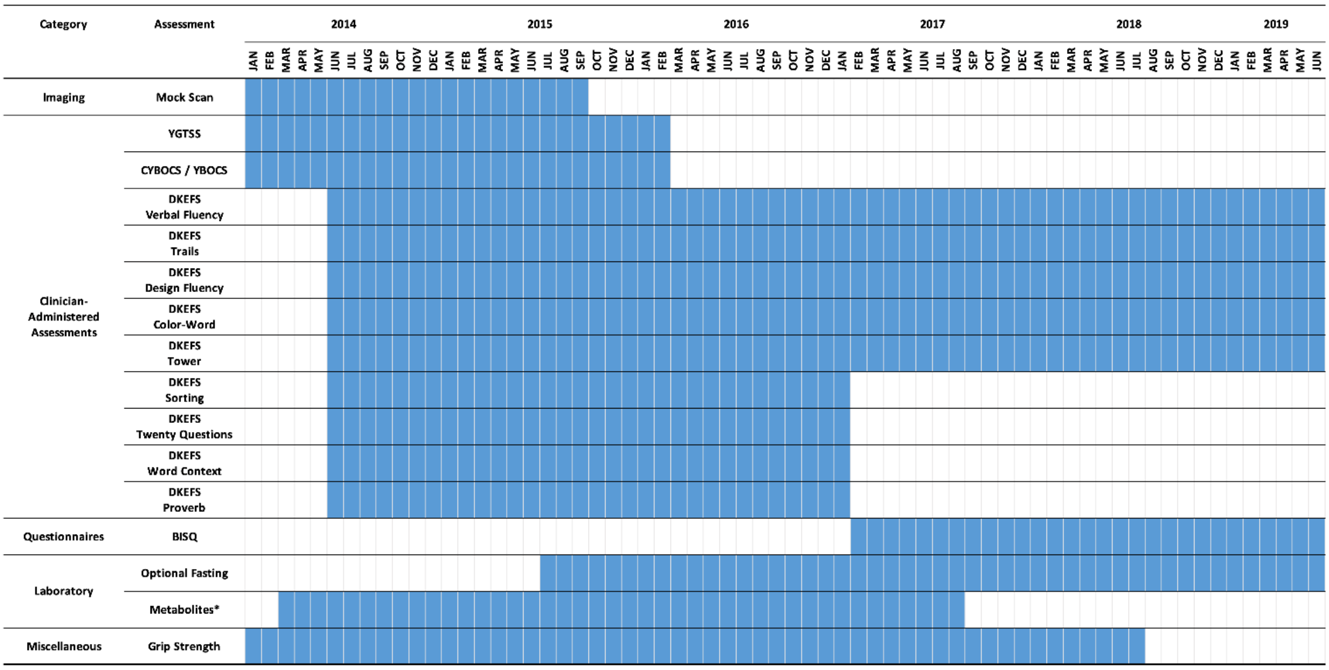

Supplementary Figure 1. Timeline of protocol modifications. Timeline of assessments that were added and/or removed from the study protocol. Baseline-only assessments identified in Table 2 were collected through the last participant enrollment in November 2017. BISQ = Brain Injury Screening Questionnaire; CY-BOCS = Children's Yale-Brown Obsessive Compulsive Scale; DKEFS = Delis-Kaplan Executive Function System; Y-BOCS = Yale-Brown Obsessive Compulsive Scale; YGTSS = Yale Global Tic Severity Scale. *Serum metabolites included isoleucine, kynurenine, leucine, phenylalanine, quinolinic acid, tryptophan, tyrosine, and valine. Analysis of serum metabolites was halted August 2017.

a

	CLG 2-Day Baseline: Track A			CLG 2-Day Baseline: Track B		
	Day 1		Day 2	Day 1		Day 2
8:30 AM	Consent		Participant Arrives/ Welcome Back	Consent		DKEFS
8:45 AM						
9:00 AM	Bloods		MRI (MRI Time: 9:00 am)	Bloods		
9:15 AM						
9:30 AM	Breakfast			Breakfast		
9:45 AM	PENN CNP	KSADS		Digit Span/WASI/WIAT	Parent Measures	
10:00 AM						
10:15 AM						
10:30 AM						
10:45 AM			MRI-Q (age 13+), MRN			CYBOCS/YGTSS
11:00 AM	KSADS			6-Minute Bike Test		MRN
11:15 AM	Mock Scan (11:00 am)			Mock Scan (11:00am)		
11:30 AM			CYBOCS, YGTSS			
11:45 AM			ANT			ANT
12:00 PM	BIRD			Family History		
12:15 PM	Family History		Dot Probe	Lunch		Dot Probe
12:30 PM	Lunch		Lunch			Lunch
12:45 PM				BIRD		
1:00 PM	Digit Span/WASI/WIAT	Parent Measures, Vineland	DKEFS	PENN CNP	KSADS	MRI (MRI Time: 1:00 pm)
1:15 PM						
1:30 PM						
1:45 PM						
2:00 PM						
2:15 PM	6-Minute Bike Test			KSADS		
2:30 PM	MRN			MRN	Parent Measures, Vineland	MRI-Q and Satisfaction Q
2:45 PM						
3:00 PM			Satisfaction Q			
3:15 PM			Debrief			Debrief

b

	CLG Baseline (9:00AM MRI TIME)		CLG Baseline (10:30AM MRI TIME)		CLG Baseline (12:30pm MRI TIME)	
8:30 AM	Re-Consent & Bloods		Re-Consent & Bloods		Re-Consent & Bloods	
8:45 AM		Parent Measures, Vineland, and Family Hx	Neuropsych		Neuropsych	Parent Measures, Vineland, and Family Hx
9:00 AM	MRI (MRI TIME: 9:00AM)			KSADS		
9:15 AM						
9:30 AM			MRN		MRN	
9:45 AM						
10:00 AM						
10:15 AM			KSADS			
10:30 AM	MRI-Q (ages 13+)	KSADS	MRI (MRI TIME: 10:30AM)		ANT	
10:45 AM	Penn CNP				Penn CNP	
11:00 AM						
11:15 AM						
11:30 AM						
11:45 AM	KSADS					
12:00 PM	Lunch		MRI-Q (ages 13+)		Lunch	
12:15 PM			Lunch			
12:30 PM	Neuropsych				MRI (MRI TIME: 12:30PM)	
12:45 PM			ANT	Parent Measures, Vineland, and Family Hx		KSADS
1:00 PM			Penn CNP			
1:15 PM						
1:30 PM	6-Minute Bike Test					
1:45 PM	MRN					
2:00 PM					MRI-Q (ages 13+)	
2:15 PM	ANT		6-Minute Bike Test		KSADS	
2:30 PM			BIRD and Dot Probe		6-Minute Bike Test	
2:45 PM	BIRD and Dot Probe				BIRD and Dot Probe	
3:00 PM			Satisfaction Q child	Satisfaction Q parent		
3:15 PM	Satisfaction Q child	Satisfaction Q parent			Satisfaction Q child	Satisfaction Q parent

c

<table>
<tr><th></th><th colspan="2">CLG Follow-Up (9:00AM MRI)</th><th colspan="2">CLG Follow-Up (10:30AM MRI)</th><th colspan="2">CLG Follow-Up (12:30AM MRI)</th></tr>
<tr><td>8:30 AM</td><td colspan="2">Re-Consent</td><td colspan="2">Re-Consent</td><td colspan="2">Re-Consent</td></tr>
<tr><td>8:45 AM</td><td rowspan="6">MRI 9:00AM)</td><td rowspan="6"></td><td colspan="2">Bloods</td><td colspan="2">Bloods</td></tr>
<tr><td>9:00 AM</td><td rowspan="3">MRN</td><td rowspan="6">KSADS</td><td rowspan="4">Digit Span/ DKEFS/ RAVLT</td><td rowspan="5">Parent Measures</td></tr>
<tr><td>9:15 AM</td></tr>
<tr><td>9:30 AM</td></tr>
<tr><td>9:45 AM</td><td rowspan="2">ANT</td></tr>
<tr><td>10:00 AM</td><td rowspan="4">Penn CNP</td></tr>
<tr><td>10:15 AM</td><td>MRI-Q (13+)</td><td rowspan="6">KSADS</td><td>KSADS</td><td></td></tr>
<tr><td>10:30 AM</td><td rowspan="4">Penn CNP</td><td rowspan="6">MRI (MRI TIME: 10:30AM)</td><td rowspan="6"></td><td rowspan="6">KSADS</td></tr>
<tr><td>10:45 AM</td></tr>
<tr><td>11:00 AM</td><td rowspan="3">MRN</td></tr>
<tr><td>11:15 AM</td></tr>
<tr><td>11:30 AM</td><td>KSADS</td></tr>
<tr><td>11:45 AM</td><td colspan="2">Bloods</td><td>KSADS</td></tr>
<tr><td>12:00 PM</td><td colspan="2" rowspan="2">Lunch</td><td>MRI-Q,</td><td></td><td colspan="2" rowspan="2">Lunch</td></tr>
<tr><td>12:15 PM</td><td colspan="2" rowspan="2">Lunch</td></tr>
<tr><td>12:30 PM</td><td rowspan="4">Digit Span/ DKEFS/ RAVLT</td><td rowspan="5">Parent Measures</td><td rowspan="6">MRI (MRI TIME: 12:30pm)</td><td rowspan="12"></td></tr>
<tr><td>12:45 PM</td><td rowspan="4">Digit Span/ DKEFS/ RAVLT</td><td rowspan="6">Parent Measures</td></tr>
<tr><td>1:00 PM</td></tr>
<tr><td>1:15 PM</td></tr>
<tr><td>1:30 PM</td><td>6-Minute Bike Test</td></tr>
<tr><td>1:45 PM</td><td rowspan="2">ANT</td><td rowspan="7"></td><td rowspan="4">Penn CNP</td></tr>
<tr><td>2:00 PM</td><td>MRI-Q,</td></tr>
<tr><td>2:15 PM</td><td rowspan="3">MRN</td><td rowspan="5"></td><td rowspan="2">ANT</td></tr>
<tr><td>2:30 PM</td></tr>
<tr><td>2:45 PM</td><td rowspan="2">BIRD and Dot Probe</td><td rowspan="2">BIRD and Dot Probe</td></tr>
<tr><td>3:00 PM</td><td rowspan="2">BIRD and Dot Probe</td></tr>
<tr><td>3:15 PM</td><td>6-Minute Bike Test</td><td>6-Minute Bike Test</td></tr>
</table>

Supplementary Figure 2. Track options for ordering of assessments. Track options include: (a) baseline two-day characterization, (b) baseline one-day characterization, and (c) follow-up visit characterization.

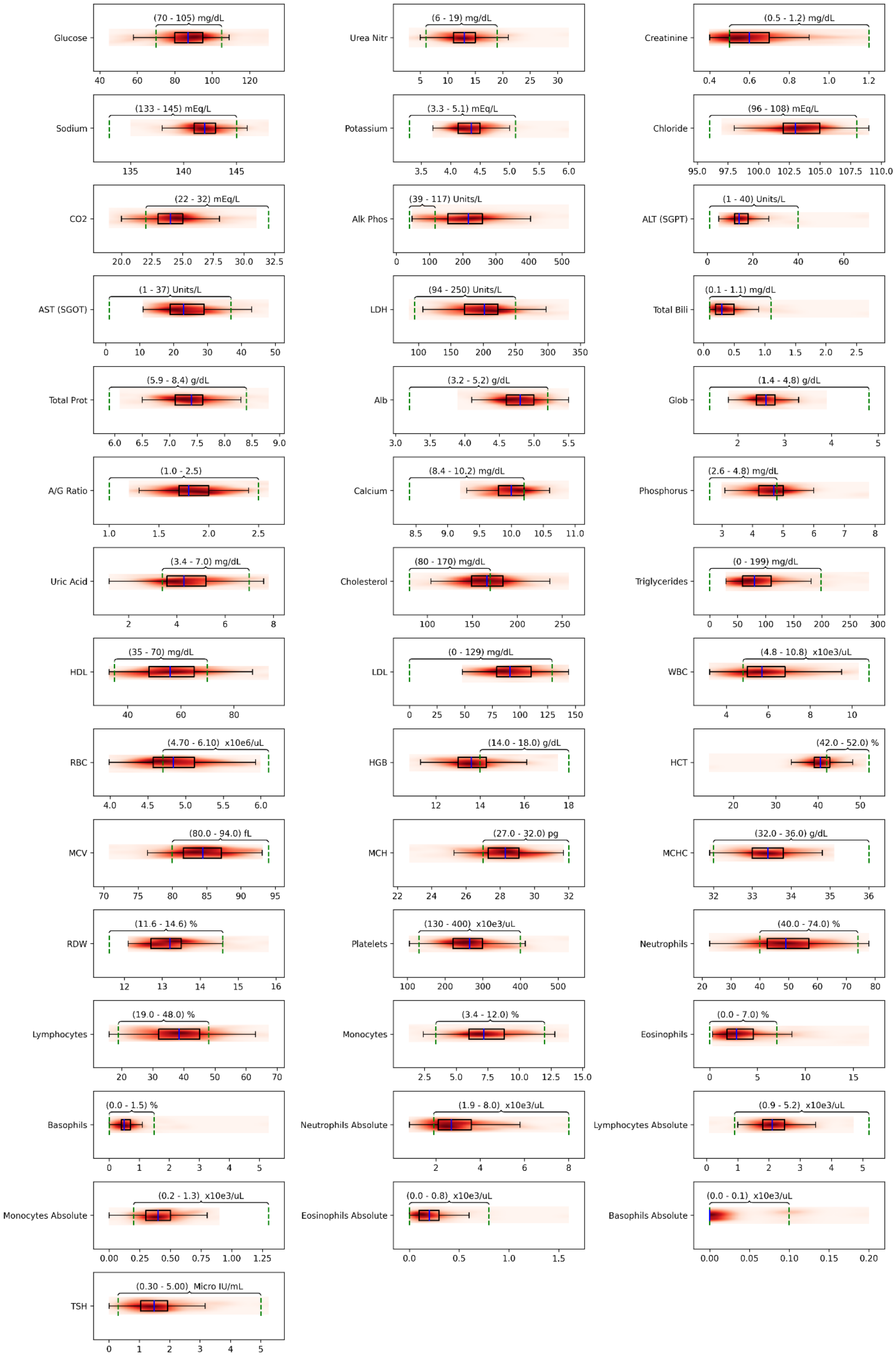

Supplementary Figure 3. Clinical laboratory test results. Box plots and density maps (deeper reds indicate more participants) are displayed for the study cohort. Laboratory reference ranges are adult-defined and displayed numerically with a horizontal bracket. Subjects were not all fasting before blood was drawn. The range of values for the Basophils Absolute for our sample is from 0.0 to 0.2, so all data are on the left side of the plot.

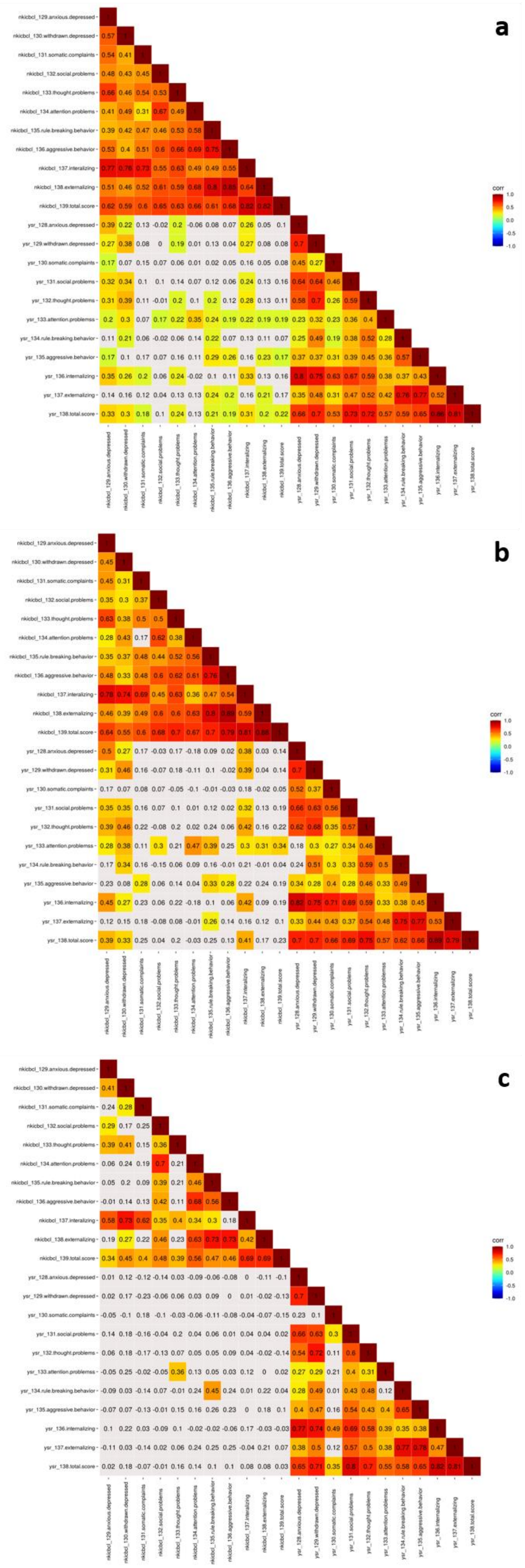

Supplementary Figure 4. Youth Self-Report (YSR) and Child Behavior Checklist (CBCL) in behavioral phenotyping. Heatmap depicting correlations between the Youth Self Report (YSR, a child report measure) and its companion parent report measure the Child Behavior Checklist (CBCL) in (a) all participants, (b) participants with any psychiatric diagnosis, and (c) participants with no diagnosis. Correlation values represented with color coding survived multiple comparisons correction (false discovery rate; q <0.05).

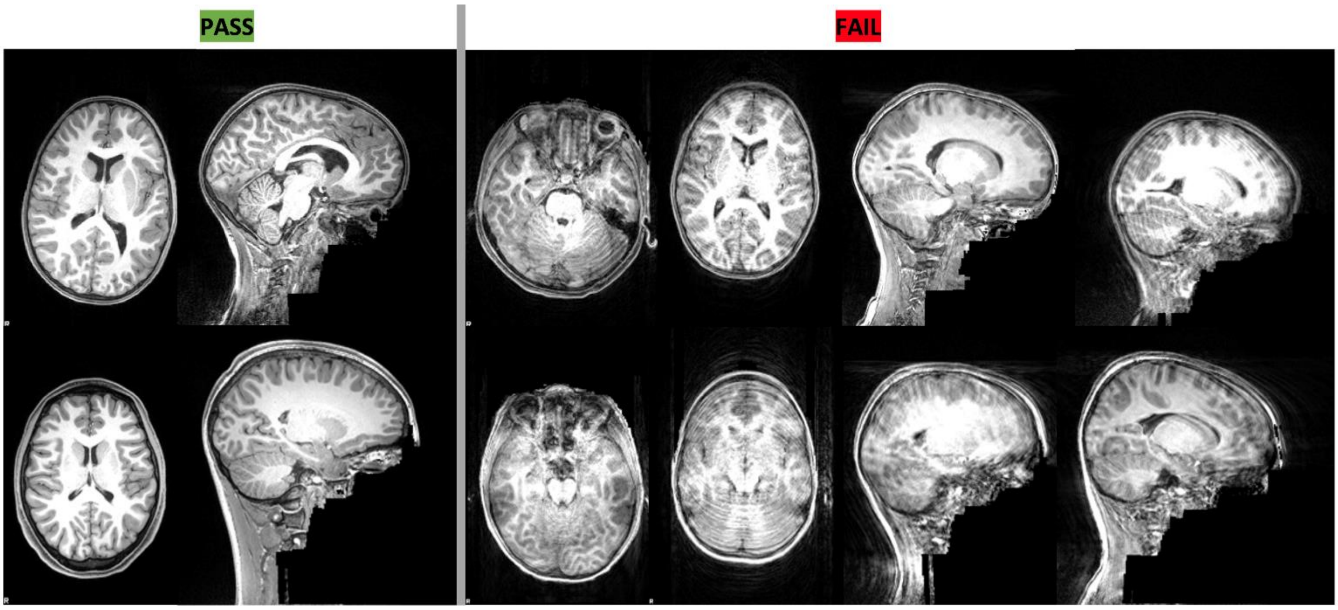

Supplementary Figure 5. Subset of example images used for training raters to pass or fail a structural image based on quality.

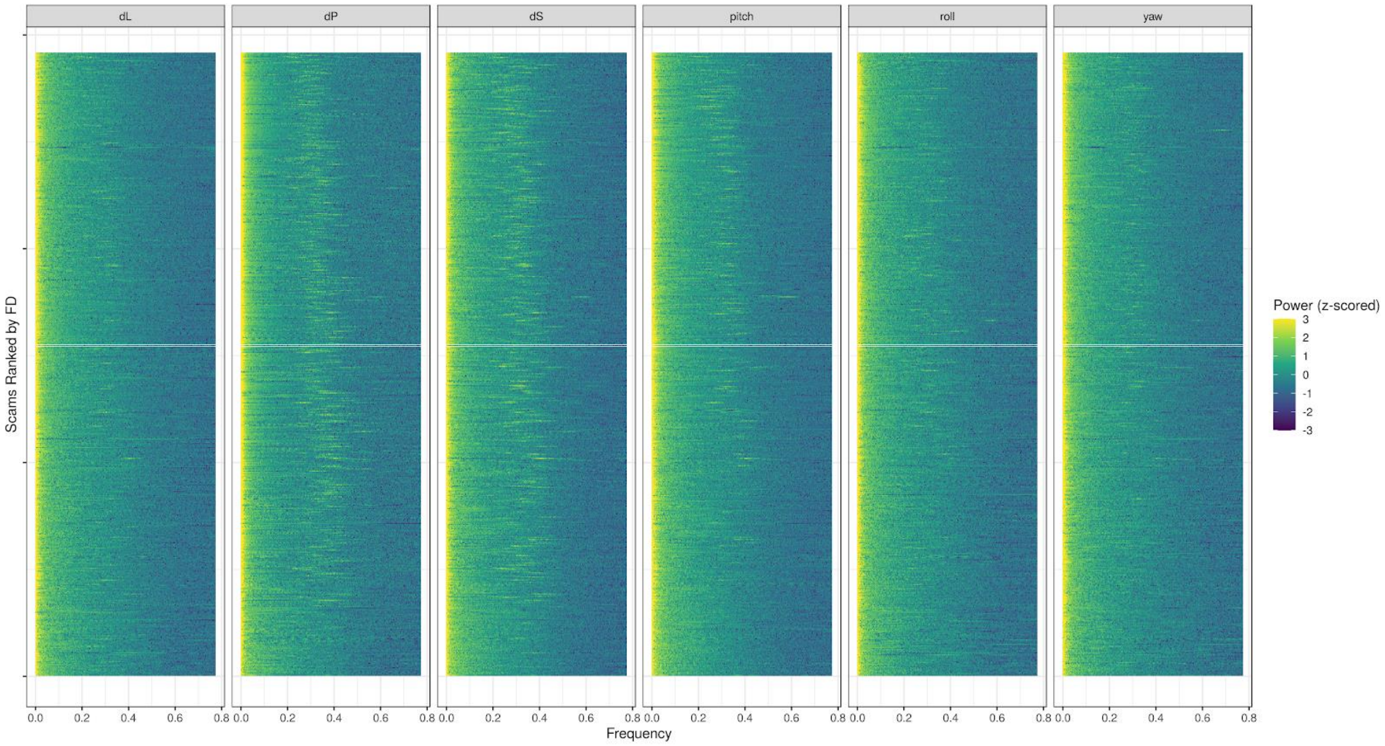

Supplementary Figure 6. Power spectrum plots of the motion parameters of the resting state scan at TR=645ms. Code used for plots was adapted from (Fair et al., 2020).

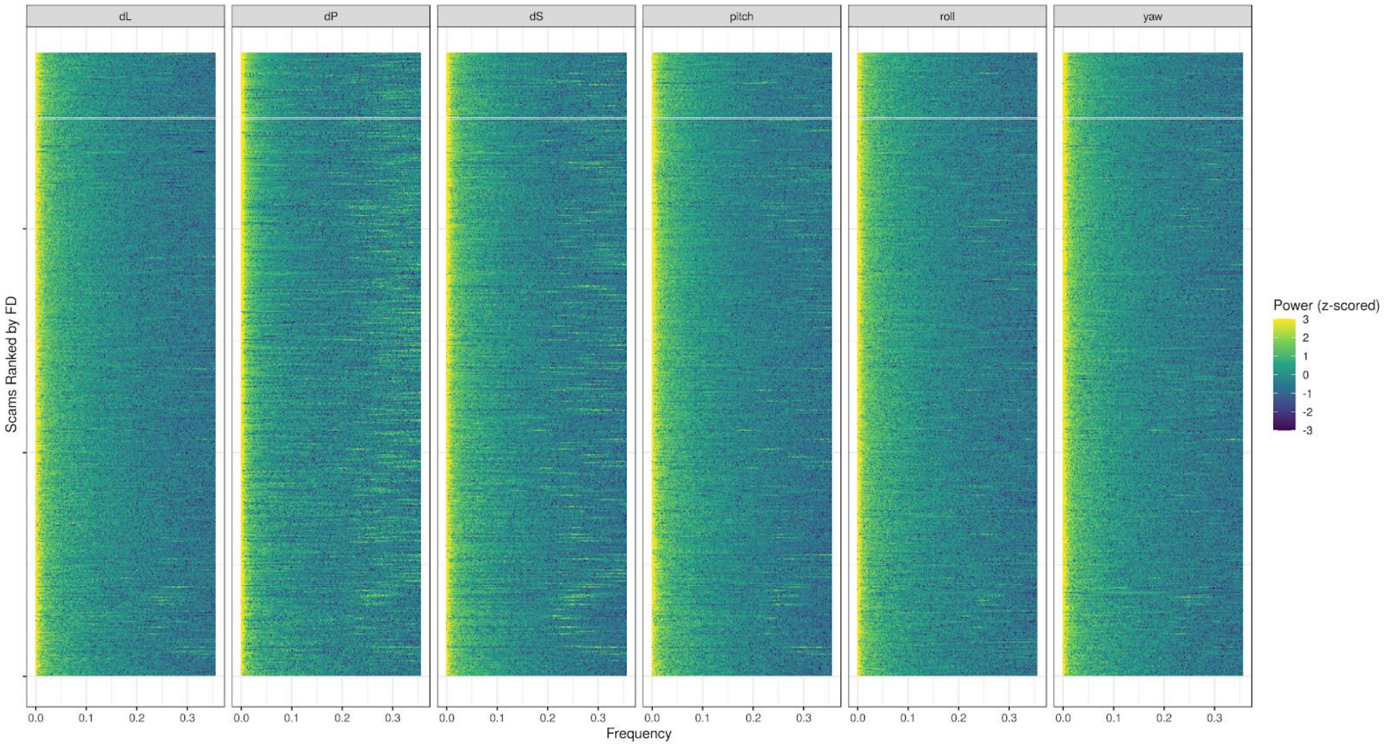

Supplementary Figure 7. Power spectrum plots of the motion parameters of the resting state scan at TR=1400ms. Code used for plots was adapted from (Fair et al., 2020).

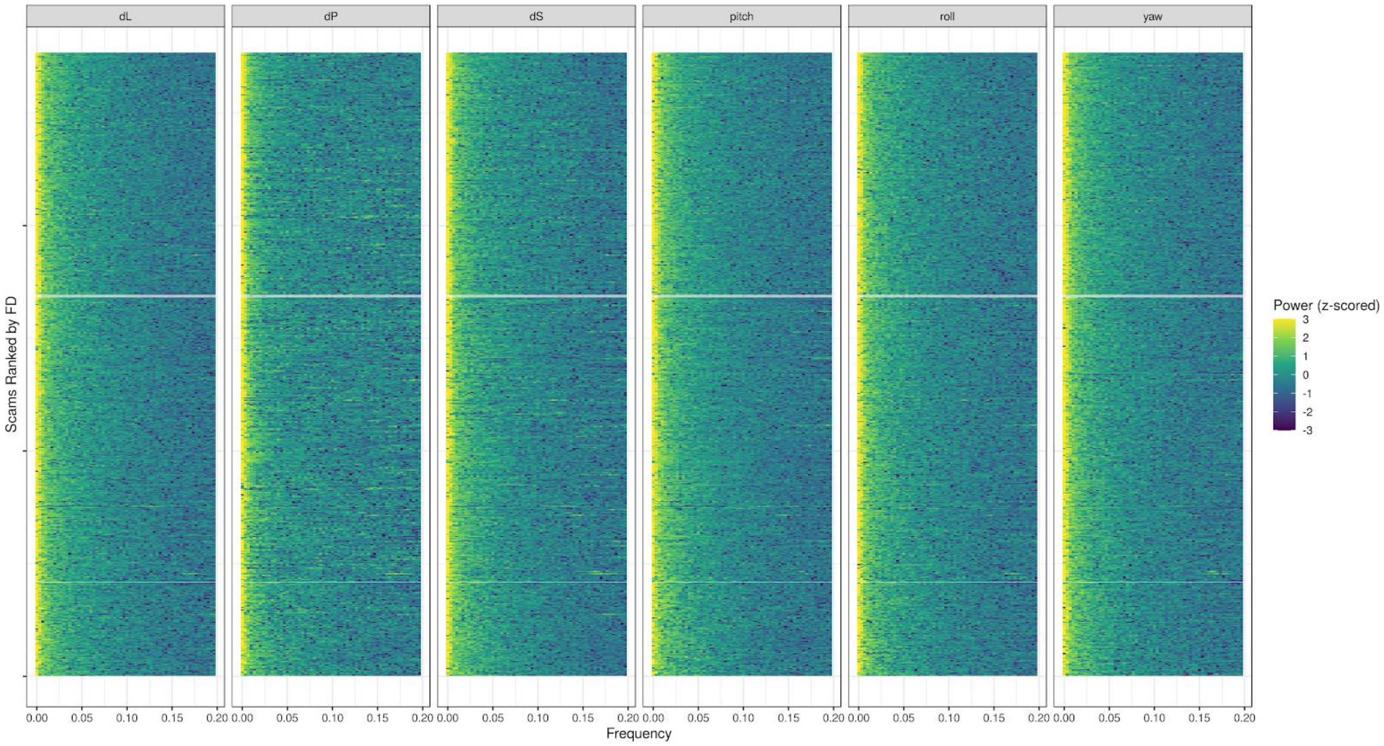

Supplementary Figure 8. Power spectrum plots of the motion parameters of the resting state scan at TR=2500ms. Code used for plots was adapted from (Fair et al., 2020).

Supplementary Tables

Referral Source	Total	%
Participant Could Not Recall	88	23.9%
Word of Mouth	82	22.3%
Flyer	44	12.0%
Prior NKI RS Participation	41	11.1%
Street Fair/Community Event	39	10.6%
NKI Neuroscience Education Day	29	7.9%
Clinical Provider Referral	19	5.2%
InfoUSA-based Mailings	13	3.5%
Email/Social Media/Online	8	2.2%
Newspaper Advertisement	3	0.8%
Radio Advertisement	2	0.5%

Supplementary Table 1. Primary referral pathways for enrolled participants.

Zip Code	Pop 2010 Census	% Pop 2010 Census	% Enrollment
10901	23,465	7.55%	5.52%
10913	5,532	1.78%	4.55%
10920	8,554	2.75%	2.60%
10923	8,732	2.81%	4.55%
10927	11,910	3.83%	3.25%
10952	38,917	12.53%	2.27%
10954	23,045	7.42%	8.12%
10956	31,521	10.15%	12.99%
10960	15,093	4.86%	9.42%
10962	5,950	1.92%	5.19%
10964	1,472	0.47%	0.32%
10965	14,791	4.76%	8.44%
10968	2,353	0.76%	1.30%
10970	9,993	3.22%	3.25%
10974	3,152	1.01%	0.32%
10976	2,258	0.73%	1.62%
10977	59,048	19.01%	4.55%
10980	13,383	4.31%	2.27%
10983	5,532	1.78%	7.14%
10984	2,842	0.91%	0.65%
10986	1,974	0.64%	0.97%
10989	9,293	2.99%	6.49%
10993	4,769	1.54%	1.95%
10994	7,085	2.28%	2.27%

Supplementary Table 2. Zip code distribution for participants residing In Rockland County.
